# The impact of clinical use on the torsional behavior of Reciproc and WaveOne instruments

**DOI:** 10.1590/1678-775720150596

**Published:** 2016

**Authors:** Rafael Rodrigues Soares de MAGALHÃES, Lígia Carolina Moreira BRAGA, Érika Sales Joviano PEREIRA, Isabella Faria da Cunha PEIXOTO, Vicente Tadeu Lopes BUONO, Maria Guiomar de Azevedo BAHIA

**Affiliations:** 1 - Universidade Federal de Minas Gerais, Faculdade de Odontologia, Departamento de Odontologia Restauradora, Belo Horizonte, MG, Brasil.; 2 - Universidade Federal da Bahia, Faculdade de Odontologia, Departamento de Clínica Odontológica, Salvador, BA, Brasil.; 3 - Universidade Federal de Minas Gerais, Faculdade de Engenharia, Departamento de Engenharia Metalúrgica e de Materiais, Belo Horizonte, MG, Brasil.

**Keywords:** Endodontics, Instrumentation, Root canal preparation

## Abstract

Torsional overload is a fracture representative parameter for instruments in single-file techniques.

**Objective:**

The aim of this study was to assess the influence of clinical use, *in vivo*, on the torsional behavior of Reciproc and WaveOne instruments considering the possibility that they degraded with use.

**Material and Methods:**

Diameter at each millimeter, pitch length, and area at 3 mm from the tip were determined for both types of instruments. Twenty-four instruments, size 25, 0.08 taper, of each system were divided into two groups (*n*=12 each): Control Group (CG), in which new Reciproc (RC) and WaveOne Primary (WO) instruments were tested in torsion until rupture based on ISO 3630-1; and Experimental Group (EG), in which each new instrument was clinically used to clean and shape the root canals of one molar. After clinical use, the instruments were analyzed using optical and scanning electron microscopy and subsequently tested in torsion until fracture. Data were analyzed using one-way analysis of variance at a=.05.

**Results:**

WO instruments showed significantly higher mean values of cross-sectional area A3 (*P*=0.000) and smaller pitch lengths than RC instruments with no statistically significant differences in the diameter at D3 (*P*=0.521). No significant differences in torsional resistance between the RC and WO new instruments (*P*=0.134) were found. The clinical use resulted in a tendency of reduction in the maximum torque of the analyzed instruments but no statistically significant difference was observed between them (*P*=0.327). During the preparation of the root canals, two fractured RC instruments and longitudinal and transversal cracks in RC and WO instruments were observed through SEM analysis.

**Conclusion:**

After clinical use, no statistically significant reduction in the torsional resistance was observed.

## INTRODUCTION

Nickel–Titanium (NiTi) rotary instruments represented significant progress in the preparation of the root canal system; however, they may experience premature failure caused by fatigue^17,24^or by torsional overload. Torsional fracture is a result of the binding of the instrument tip inside the root canal, which is typically smaller than the instrument tip diameter^20^. The rupture occurs when the torsional stress locks the instrument in dentine walls and becomes higher than the maximum torque; the instrument can withstand at that point^15^. This risk may be reduced by performing coronal enlargement and by creating a glide path before using any NiTi rotary instrument^3^.

Two brands of NiTi instruments, adopting the single-file system and advocating the reciprocation concept, were recently introduced into the market: Reciproc (VDW, Munich, Germany) and WaveOne (Dentsply-Maillefer, Ballaigues, Switzerland). These files are made of a NiTi alloy called M-Wire that is produced by an innovative thermomechanical treatment process^12,14,18,19^. They employ a reciprocating motion, rather than a continuous rotary motion; the former reduces the torsional stress by periodically reversing the rotation, and ultimately increases the lifespan of the instrument^14,27^. In the reciprocating motion, the values of clockwise and counterclockwise rotations are different for each instrument. A large rotating angle, counterclockwise (CCW) to the cutting direction, determines that the instrument advances in the canal to engage and cut the dentin, whereas a smaller angle, clockwise (CW) to the cutting direction, allows the file to be immediately disengaged and safely progresses along the canal path, while reducing the screwing effect and file separation^22^.

Previous studies showed that the reciprocating instruments promoted minimal apical transportation, maintaining the original canal curvature better than rotary instruments, although they are associated with producing higher debris extrusion^4,6^.

Several studies assumed that the reciprocating motion improved flexural fatigue resistance in NiTi instruments, when compared with continuous rotary motion^9,13,15,16,22^. Kim, et al.^14^ (2012) studied flexural fatigue and torsional resistance and demonstrated that Reciproc had the longest fatigue life while WaveOne presented the highest torsional resistance. The same outcome for WaveOne was also demonstrated in another study by Elnaghy and Elsaka^8^ (2015), while Ha, et al.^10^ (2015) found no difference in torsional resistance comparing Reciproc and WaveOne instruments.

However, very limited information is available on the torsional behavior of instruments used in single-file systems in reciprocating motion. Each time a NiTi instrument encounters resistance it undergoes torsional loading. The load is greater whenever the dentin is hard or the canal diameter is small. This torsional load exerted on the surface of the instrument can prevent its rotation to a greater or lesser extent. In extreme cases, the instrument may fracture when the resistance is so high that it constrains the movement of the instrument^2^.

To evaluate the magnitude of the torsional load to which the instruments are subjected when used in single-file techniques is of fundamental importance. Considering that the instrument progress in the canal without pre-enlargement and being aware that the amount of torque generated during the shaping of root canals clearly depends on the size of the contact areas between the instruments and the canal walls^20^, the purpose of this research was to analyze the effect of the clinical use on torsional resistance of Reciproc and WaveOne instruments.

## MATERIAL AND METHODS

The instruments employed in this study were Reciproc (RC/R25) (VDW GmbH, Munich, Germany), and WaveOne (WO/Primary) (Dentsply-Maillefer, Ballaigues, Switzerland) both types size 25, 0.08 taper.

Before the mechanical testing, all the brand new instruments received from the manufacturers were photographed using a high-resolution digital camera (20D; Canon, Tokyo, Japan). Their dimensional characteristics were assessed against the American National Standards Institute/American Dental Association Specification No. 101. The measurements (*n*=12 for each type) were obtained using Image Pro Plus 6.0 (Media Cybernetics; Silver Spring, MD). Lines were drawn on both sides of the file’s images, and the outermost diameters at each millimeter from the tip were measured. The same method was used to determine the pitch length. To visualize and measure the cross-sectional area at 3 mm from the tip (A3), three instruments of each type were randomly selected and cut to approximately 2.7 mm from the tip using a metallographic cutter (Isomet 1000; Buehler, Illinois, USA). The cross-sectional surfaces were polished with sandpaper to 3.0 mm from the tip and then imaged using a scanning electron microscope (SEM) (JSM 6360; Jeol, Tokyo, Japan) at 150× magnification. The cross-sectional areas for each instrument were determined using the same software described above. Such dimensional and geometrical characteristics are important in this study and may have influence on torsional behavior^16^. Three instruments of each system were examined by SEM to assess their surface characteristics verifying if there were surface defects, such as microcracks, even in new instruments.

The instruments were then divided into two groups, each one containing twelve new instruments of each type: (i) Control Group (CG), in which a torsion test was performed to establish the mean values of maximum torque and angular deflection at fracture, and (ii) Experimental Group (EG), in which the instruments were employed clinically, *in vivo,* by an experienced endodontist to shape the root canals (3 or 4 *per* tooth) of one upper or lower molar randomly distributed between the two groups of instruments. After the orifices were located and the canal explored with stainless steel K-files (sizes #10 and #15) (Dentsply-Maillefer, Ballaigues, Switzerland), patency was established. Direct and angled radiographs of each tooth were obtained using a paralleling technique to evaluate anatomy, as well as to determine the canal radius and angle of curvature, as defined by Pruett, et al.^23^ (1997), and its approximate length. These parameters were measured by projecting the radiographic images using a profile projector (Mitutoyo, Tokyo, Japan) at 10× magnification. The canal radius of curvature was measured along the outer canal wall.

Then, the canals were cleaned and shaped in accordance with the manufacturer’s instructions. The instrumentation was completed gently with an “in-and-out” motion, and each canal was prepared until the working length was reached (0.5 mm of the canal patency length), when the instrument was immediately withdrawn. The instruments were operated using the manufacturer’s pre-programmed settings for a Silver Reciproc engine (VDW GmbH, Munich, Germany): “Reciproc all” mode for RC size 25 and “WaveOne all” mode for WO Primary. The instrument was removed from the canal after three pecks or when resistance was encountered, and it was used in a lateral brushing motion. A 5.25% sodium hypochlorite solution was used for irrigation and RC-Prep (Premier Dental Products, Norristown, PA, USA) was used as a lubricant. The flutes of the instrument were cleaned after each group of the three “in-and-out” movements.

After use in each patient, the instruments were washed and ultrasonically cleaned for 5 minutes in acetone. The RC and WO instruments of the EG were observed by optical microscopy (Mitutoyo TM, Tokyo, Japan), at 30× magnification, to determine the presence of distortion, unwinding defects, and any macroscopic deformation. Before the torsion test, the same three instruments of each system, previously examined by SEM, were reexamined in the same position and area to assess the effect of the clinical use on their surface characteristics.

The torsion tests were performed based on ISO 3630-1 specification^11^ using a torsion machine described in detail elsewhere^1^. Torque values were assessed by measuring the force exerted on a small load cell by a lever arm linked to the torsion axis. A resistive angular transducer connected to a process controller measured and controlled the rotation angle. The rotation speed was set clockwise to 2 rpm. The end of the shaft was clamped into a chuck that was connected to a reversible geared motor. Three millimeters of the instrument’s tip was clamped into another chuck with brass jaws to prevent it from sliding. Continuous recordings of torque and angular deflection, as well as measurements of the maximum torque and angular deflection at failure, were provided by a specially designed computer program. To determine the statistically significant difference in the measured parameters amongst the different groups, data obtained were subjected to a one-way analysis of variance (Anova). Significance was determined at the 95% confidence level.

## RESULTS

**Figure 1:**
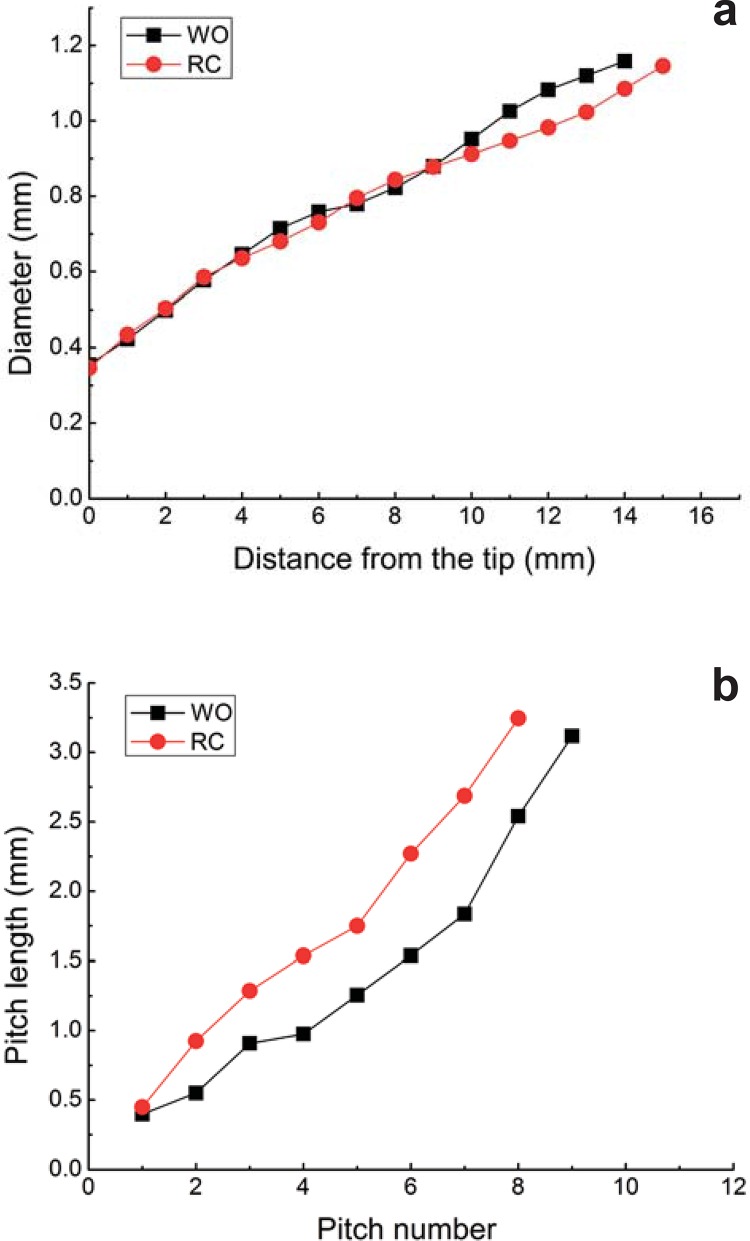
(a) Mean values of diameter (mm) as a function of distance (mm) from the tip; (b) Mean values of pitch length (mm) as a function of pitch number

The mean values (± standard deviations) of diameter at 3 mm from the tip (D3) for RC and WO were 0.504±0.010 mm and 0.499±0.020 mm, respectively (Figure 1a). Moreover, the pitch length increased along the active part of both instruments, with a steeper increase recorded in RC instruments. In general, RC presented larger pitch lengths than WO instruments, as illustrated in Figure 1b.

In this study, both file systems used had different cross-sections, with RC presenting an S-shaped cross-section and WO presenting a concave triangular-shaped cross-section. Similarly, the mean values of area at 3 mm from the tip, A3, for RC and WO were 0.112±0.005 mm^2^ and 0.123±0.005 mm^2^, respectively. However, although no statistically significant differences regarding the D3 (*P*=0.521) were present, WO instruments showed significantly higher mean values of A3 than RC (*P*=0.000).

**Figure 2:**
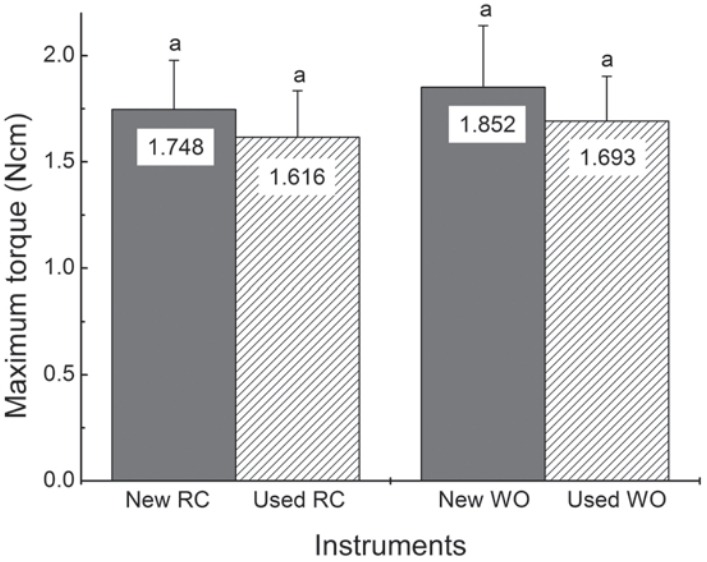
Mean values of maximum torque in torsion tested new and used RC and WO instruments (n=12 for each type); standard deviations shown as error bars. Bar values marked with the same letter were not statistically different (P>0.05)

The mean values of maximum torque determined for RC and WO new instruments (CG) were 1.763±0.226 Ncm and 1.852±0.293 Ncm, respectively (Figure 2). Although new WO instruments showed higher values for maximum torque than RC instruments, no statistically significant difference was observed between them (*P*=0.409). The angular deflection values were 295±35° and 241±28° for RC and WO instruments, respectively, with a statistically significant difference (*P*=0.000).

Radius and angle of curvature mean values (± standard deviations) characterizing the geometry of the root canals of RC and WO instruments were 5.3±1.9 mm; 5.5±2.2 mm and 23±11°; 24±15°, respectively. One-way ANOVA showed no significant difference (*P*>0.05) in root canal geometry among the groups.

The mean values of torsional resistance after clinical use (EG) are also summarized in Figure 2. The graphs illustrated a decrease in torsional strength for both instruments evaluated. However, statistical analysis of the maximum torque values for the RC and WO new instruments and after clinical use, revealed no significant difference (*P*=0.072 and *P*=0.147, respectively). The angular deflection values decreased after clinical use, except for RC instruments. However, statistical analysis showed no significant differences in this parameter between pairs of instruments examined: new WO x used WO (*P*=0.067) and new RC x used RC (*P*=0.192).

**Figure 3:**
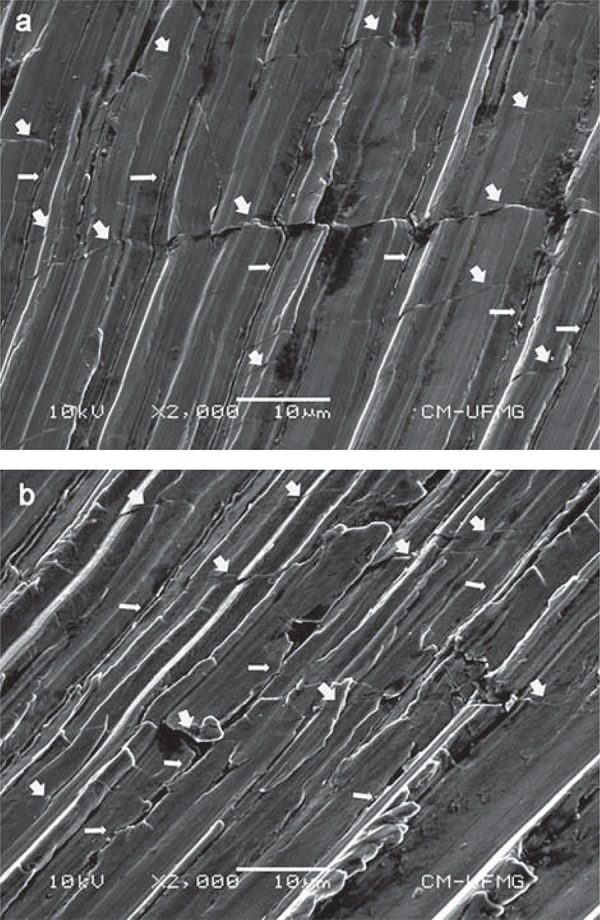
Longitudinal/parallel (wide arrows) and transverse (thin arrows) cracks in Reciproc (a) and WaveOne (b) instruments, respectively, subjected to clinical use

As illustrated in Figure 3, the lateral surfaces of all RC and WO instruments submitted to root canal shaping exhibited longitudinal microcracks, i.e., cracks parallel to the longitudinal axis of the instrument. In addition, transversal microcracks along the cutting edge were observed in the instruments after clinical use. From these images, it is noteworthy that both instruments presented similar crack patterns. A predominance of larger and wider cracks in the region between 1.0 and 2.5 mm from the instrument tip was observed.

**Figure 4:**
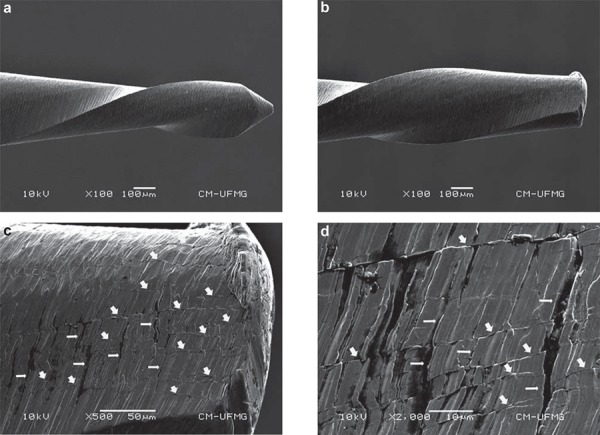
Secondary electron images obtained by scanning electron microscopy. New Reciproc (a) and fractured Reciproc after clinical use (b). Presence of high concentration of typical longitudinal/parallel (wide arrows) and transverse (thin arrows) microcracks on the surface of a RC instrument fractured during clinical use (c, d)

Figure 3 reveals a high concentration of typical transversal and longitudinal microcracks on the surface of a RC instrument that fractured during clinical use. In this investigation, two RC instruments experienced intracanal failure during clinical use (Figure 4).

## DISCUSSION

Several factors, such as anatomy of the root canals, size, taper, design, alloy chemical composition, and thermomechanical process applied during manufacturing might affect the torsional behavior of NiTi instruments^15^. No significant difference in radius and angle of curvature could be found among the teeth and thus the influence of geometrical factors on the torsional behavior was avoided.

It is important to emphasize the dimensions of the instruments at 3 mm from the tip because this is the point of apprehension of the instruments for torsional tests (according to ISO 3630-1), and where the maximum curvature is found in most root canals in clinical practice with the greatest mechanical solicitations^26^. Although similar diameters at D3 were presented, WO had a mean A3 significantly higher (*P*=0.000) than the one observed for RC instruments. As discussed in the literature, the maximum torque values increased proportionally with A3^15^. It is also generally accepted that the larger the pitch length, the smaller is the torsional resistance of an endodontic instrument^7^. WO instruments presented smaller pitch length than RC instruments. These characteristics of smaller pitch length and higher A3 lead to a tendency of a higher torsional strength for WO instruments. These results corroborate a recent clinical trial that accessed the fracture incidence of the reciprocating WaveOne file when used to prepare root canals of posterior teeth. The overall instrument separation incidence concerning the number of canals shaped was found to be as low as 0.13%^5^.

Although these instruments are less susceptible to the “taper lock” effect due to the clockwise motion of detachment, once used as a single-file technique without any pre-flaring, they can operate under higher torsional loads owing to increased contact area with dentin walls, which may exceed its torsional strength and eventually lead to fracture^2,20^. The clinical application caused a tendency of decreasing the maximum torque but consumed less than 10% of the torsional resistance of the RC and WO instruments. Compared with the new instruments, no statistically significant difference in the torsional resistance was observed after the clinical use (*P*>0.05). It was previously reported that clinical use in 10 curved canals or 5 molars, lowered the mean values of maximum torque compared with the new instruments^1,25,26^. This significant decrease in torsional resistance was not observed in this study and may be explained by the reduced clinical use (only 1 molar) or by the reciprocating motion of these instruments. The angular deflection mean values obtained by RC (295°) and WO (241°) instruments were far beyond the amplitude of rotation employed in the CCW movement (170°/150°), which may also explain the low incidence of failure during the clinical use. In a recent study, a very low incidence of fracture in Reciproc instruments and deformation after clinical use was also observed^21^. According to the literature, this low frequency of fracture can be possibly understood as an improvement in fatigue resistance associated with the reciprocation kinematics^16^, the instrument cross-sectional design^22^, and the superelastic M-wire alloy^12,18,19^.

In accordance with the manufacturer and during the clinical use, these instruments were used under apical pressure. Longitudinal cracks in RC and WO instruments (Figure 3) reflecting the stress orientation on their surfaces under torsional load were observed through SEM analysis. According to Bahia, et al.^2^ (2008), during cyclic torsion, planes with a maximum shear stress are either perpendicular and/or parallel to the longitudinal axis indicating that instrument fracture may occur as a result of tri-axial stresses, which could be the case in fractured RC instruments that presented both types of cracks (Figure 4). Nevertheless, the fracture that occurred in both RC instruments may be explained by the lower mean A3 values and the larger pitch length in comparison with WO instruments, thus promoting a smaller mass at the tip of the RC instruments.

## CONCLUSIONS

Within the limitations of this study, no statistically significant differences in torsional resistance between Reciproc and WaveOne instruments were observed, although data suggest a tendency to higher torsional strength for WaveOne instruments. Geometric factors such as the pitch length, the diameter, and area at 3 mm from the tip, in addition to the manufacturing process, may have an impact on the clinical behavior of these instruments. Finally, we observed that the clinical use in one molar presented no statistically significant reduction in the torsional resistance for the analyzed instruments.
